# The Value of a Second Opinion for Breast Cancer Patients Referred to a National Cancer Institute (NCI)-Designated Cancer Center with a Multidisciplinary Breast Tumor Board

**DOI:** 10.1245/s10434-018-6599-y

**Published:** 2018-07-03

**Authors:** Denise Garcia, Laura S. Spruill, Abid Irshad, Jennifer Wood, Denise Kepecs, Nancy Klauber-DeMore

**Affiliations:** 1 Department of Surgery, Medical University of South Carolina, Charleston, SC USA; 2 Department of Pathology, Medical University of South Carolina, Charleston, SC USA; 3 Department of Radiology, Medical University of South Carolina, Charleston, SC USA; 4 Hollings Cancer Center, Medical University of South Carolina, Charleston, SC USA

## Abstract

**Background:**

This study aimed to investigate the changes in diagnosis after a second opinion for breast cancer patients from a multi-disciplinary tumor board (MTB) review at an National Cancer Institute (NCI)-designated cancer center.

**Methods:**

A retrospective study analyzed patients with a breast cancer diagnosed at an outside institution who presented for a second opinion from August 2015 to March 2016 at the Medical University of South Carolina (MUSC). Radiology, pathology, and genetic testing reports from outside institutions were compared with reports generated after an MTB review and subsequent workup at MUSC. The second-opinion cases were categorized based on whether diagnostic variations were present or not.

**Results:**

The review included 70 patients seeking second opinions, and 33 (47.1%) of these patients had additional radiologic images. A total of 30 additional biopsies were performed for 25 patients, with new cancers identified in 16 patients. Overall, 16 (22.8%) of the 70 of patients had additional cancers diagnosed. For 14 (20%) of the 70 patients, a second opinion led to a change in pathology interpretation. Genetic testing was performed for 11 patients (15.7%) who met the National Comprehensive Cancer Network (NCCN) guidelines for genetic testing, but none showed a mutation other than a variant of unknown significance. After a complete workup, 30 (42.8%) of the 70 patients had a change in diagnosis as a result of the MTB review.

**Conclusion:**

A review by an MTB at an NCI-designated cancer center changed the diagnosis for 43% of the patients who presented for a second opinion for breast cancer. The study findings support the conclusion that referral for a second opinion is beneficial and has a diagnostic impact for many patients.

Surgical second-opinion programs were developed in the 1970s, and since that time have been assessed for cost efficiency and improvement of patient care.^1^ The popularity of second-opinion programs increased into the 1980s.^2^ Several programs demonstrated a cost efficacy benefit, especially in avoidance of unnecessary procedures.^3^ One early study of second-opinion programs that focused on prostate cancer patients showed that these programs could save money by preventing unnecessary surgeries as a result of pathology found to be benign at a second-opinion review.^4^ These early second-opinion programs came into question during the 1990s due to recognition that no definitive way existed to assess which proposed surgical treatment was correct.^5^

One major flaw in second-opinion studies is that no “gold standard” exists for pathology to assess whether the first or second opinion is correct. Additionally, should a difference in pathology from benign to malignant be ascertained on a second opinion, the increase in cost for further evaluation and management still would be beneficial and warranted.^6^

Despite some critiques, studies have continued to be published with results that support the benefit of second opinions in surgery. One paper reviewing prostate cancer assessed 535 cases and found that even with a small number of changes in diagnosis (1.3%), thousands of dollars were saved by preventing needless operations.^4^ More recently, a study from the University of Iowa found a 2.3% rate for “major diagnostic disagreements” on second-opinion review of surgical pathology.^6^

In the past few decades, tertiary care centers have started implementing multi-disciplinary tumor boards (MTBs) to evaluate cancer cases. One of the earliest studies investigating the efficacy of MTBs was from the University of Pennsylvania, which had a high percentage (43%) of cases in which their MTB disagreed with the management planned by the outside provider. Additionally, 31% of the patients reviewed by the MTB underwent further workup.^7^

As they have gained popularity, MTBs have been implemented nationwide and are becoming the standard of care in cancer centers. Regardless of widespread acceptance, studies and reviews assessing the efficacy and utility of MTBs continue to warrant investigation. A recent study reviewed 200 cases, finding new cancers detected in 5% of patients.^8^ Additionally, in 4% of the cases, they reported avoidance of biopsies deemed unnecessary. Findings have shown MTBs to be efficacious in identifying additional cancers and also preventing unnecessary harm. Studies specifically addressing MTBs’ evaluation of second opinions also have resulted in changes in diagnoses and patient management.^8^

Multi-disciplinary tumor boards have been developed internationally and are becoming the standard of care in cancer centers. Typically, they involve meetings between specialists within a specific cancer field such as breast cancer and can include radiation oncologists, surgical oncologists, medical oncologists, pathologists, and radiologists.^9^ One large review of 5629 MTB second-opinion cases involved several cancers during a 3-year period. Only a small percentage (2.3%) of the cases resulted in major diagnostic disagreements, defined as those requiring changes in clinical management. Interestingly, in this study, none of the major diagnostic disagreements involved breast cancer management.^6^

Another more recent study by Charara et al.^10^ in 2017 focused more on the decision-making process involved in an MTB. However, of 503 cases presented, only 12 resulted in a change in radiology findings, and only 6 cases had a change in pathology. Although this study did not focus on second opinions, it was noted in the paper that half of the patients in each of the radiology and pathology change groups had second opinions.^10^

Despite the documented benefit of MTB review, many patients with breast cancer receive care without tumor board review at non–NCI-designated cancer centers. This study evaluated the change in radiology and pathology diagnosis and referral for genetic counseling as a result of this intervention.

## Methods

The Institutional Review Board at the Medical University of South Carolina (MUSC) approved this study. We performed a retrospective observational analysis of patients who presented to the MUSC Hollings Cancer Center for evaluation from August 2015 to March 2016 with a diagnosis of stages 0–3 breast cancer. This review included only patients with a diagnosis determined outside MUSC who were referred or self-referred for a second opinion.

All second-opinion patients had pathology slides and radiologic imaging reviewed by fellowship-trained breast radiologists and pathologists and were presented at an MTB. The breast cancer MTB at MUSC is a weekly collaborative meeting of surgical oncologists, medical oncologists, radiation oncologists, radiologists, pathologists, genetic counselor, and nurse navigators. The physician to whom the patient was referred, typically the surgical oncologist, presents the patient.

Zip codes were obtained, and using the Health Resources & Services Administration database,^11^ we were able to stratify our patient population into the lowest and highest health care-funded counties in South Carolina. The radiology reports were reviewed to ascertain whether the first opinion was obtained at an academic medical center (a hospital affiliated with a medical school).

We compared the outside radiology reports with the reports generated by MUSC radiologists. Changes in diagnosis as a result of additional assessment or imaging were noted. This included additional imaging recommended, additional biopsies recommended, and additional new cancer diagnosed in the ipsilateral or contralateral breast or axillary lymph node. Change in pathologic diagnosis was assessed by comparing the outside pathology report with the MUSC pathology report. The recommendation for genetic counseling was obtained from the outside physicians’ notes and compared with the review at our MTB to determine whether the patient met the NCCN guidelines for genetic testing or not.

### Radiology Review

Outside films were reviewed in a radiology consensus conference at MUSC that consisted of board-certified radiologists, all of whom had completed fellowship training in breast radiology. When necessary, additional views were recommended and ordered, including mammograms, breast or axillary ultrasound, or breast magnetic resonance imaging (MRI). Based on this review, some patients were scheduled to undergo stereotactic core biopsy, ultrasound-guided core biopsy of the breast or axilla, or MRI-guided biopsy (of either the ipsilateral or contralateral breast). The parameters analyzed for radiology were change in tumor size, new lesion, new calcifications, additional views recommended (mammogram, breast ultrasound, axillary ultrasound, breast MRI), additional biopsy performed (stereotactic, breast ultrasound, axillary ultrasound, or MRI-guided biopsy), additional cancer diagnosed in the ipsilateral breast, additional contralateral breast cancer diagnoses, and new axillary metastases identified.

### Pathology Review

Pathology was reviewed by board-certified pathologists with specialization in breast cancer. Hormone receptors for estrogen, progesterone, and human epidermal growth factor 2 (HER2)neu were evaluated but not routinely repeated unless the internal control was negative or unless HER2neu was equivocal. The data for pathology were assessed for change in histology, change in tumor grade, change in tumor size, and change in ER, PR or HER2/neu status.

### Genetic Testing Review

All the patients were reviewed by the tumor board to determine whether a referral for genetic counseling was indicated. We identified the number of patients who met the NCCN guidelines for genetic testing but did not have this offered at the initial institution. Patients who saw the genetic counselor at MUSC and met the NCCN guidelines for genetic testing were offered genetic testing.

## Results

The review of charts from August 2015 to March 2016 found that 70 (31%) of the 295 patients had their diagnosis determined outside MUSC and presented for a second opinion. Additional radiology recommendations for additional imaging or biopsy were made to 43 patients (61.4%) after MTB radiology assessment. The pathology second-opinion review showed a change in diagnosis for 14 (20%) of the 70 patients. According to the review, 11 (15.7%) of the 70 patients were referred for genetic testing at MUSC based on the NCCN guidelines, but genetic testing was not recommended at the outside institution.

Using the Health Resources & Services Administration database, we identified that 33% of all the patients presenting for a second opinion came from medically underserved areas. Only 6 of the 70 patients had their first opinion from another academic medical center (3 from South Carolina and 3 from other states).

### Radiology

After the MTB review, 43 (61%) of the 70 second-opinion patients had additional calcifications or lesions found, additional images obtained, or additional breast or axillary biopsies recommended. Of those 43 patients with extra imaging, 16 had an additional ipsilateral, contralateral, or axillary lymph node metastases found. This population represented 22.9% of all the second-opinion patients (Table 1).

**Table 1:** Details of the changes made after multi-disciplinary tumor board radiology review

Category	Patients *n* (%)
Additional imaging	33/70 (47)
Additional biopsy	30/70 (43)
Any additional breast cancer diagnosed	16/70 (23)
New lymph node metastasis diagnosed	5/70 (7)
Additional calcifications identified	8/70 (11)
Change in radiographic tumor size	11/70 (16)

Additional forms of imaging were obtained by 33 second-opinion patients after the MTB review, and several had more than one imaging method used. Of the 33 patients, 11 (33.3%) underwent a supplementary breast MRI, 13 (39.4%) had an additional breast ultrasound, 12 (36.4%) had an additional axillary ultrasound, and 8 (24.2%) underwent additional mammography (Table 2).

**Table 2:** Type of additional imaging obtained

Types of additional imaging	Patients	Percentage of patients having additional imaging (*n* = 33)
Breast MRI	11	33.3
Breast ultrasound	13	39.4
Axillary ultrasound	12	36.4
Mammogram	8	24.2

*MRI* magnetic resonance imaging

The review showed 30 additional biopsies for 25 patients. Of the 25 patients who had additional biopsies, 16 (64%) resulted in discovery of an additional cancer, which accounted for about 23% of the 70 patients presenting for a second opinion (Table 3).

**Table 3:** Percentage of additional biopsies that resulted in cancer

Additional biopsies	No. of biopsies	Patients positive for cancer *n* (%)
Ipsilateral breast	18	10/70 (14)
Contralateral breast	5	1/70 (1)
Axillary node	7	5/70 (7)
Total	30	16/70(23)

### Pathology

Changes in pathology represented a smaller number of variations (14/70, 20%) within the second opinions (Table 4). The most frequent variation was a change in histology (10%). The pathologic variations included changes from invasive to noninvasive cancer and, in one instance, a change from sarcoma to malignant phyllodes tumor. No changes were observed in the review of slides for ER, PR, or HER2neu staining.

**Table 4:** Changes in pathologic diagnosis

	Patients with a change in pathology *n* (%)
Change in tumor grade	4/70 (6)
Change in histology	7/70 (10)
Invasive to in situ carcinoma	1/70 (1)
Change in tumor size	2/70 (3)
Total pathology variations	14/70 (20)

### Genetics

Of the 70 patients seeking a second opinion, 11 (15.7%) met the NCCN guidelines for genetic testing but had not been offered testing before their second opinion. Of these 11 patients, 2 were found to have variants of unknown significance (VUS), which did not change the management of their disease. The one patient had a VUS in MSH6, and the other patient had a VUS in BMPR1A.

## Discussion

Additional imaging and biopsies resulted in 23% of the patients receiving a diagnosis of either a new ipsilateral or contralateral cancer or axillary metastasis. The net number of patients in our study who experienced a change in new cancers or a pathology change in diagnosis, excluding overlapping patients, was 30 (43%) of the 70 patients. The results of this observational study suggest that an MTB-assessed second opinion in a breast cancer case at an NCI-designated facility is valuable for a thorough workup for breast cancer patients.

Other reports evaluating a change in diagnosis for patients with breast cancer who sought a second opinion showed a lower percentage of diagnostic changes than our studies. In the reported literature, the rate of changes in diagnosis ranges from 0 to 13% depending on the cancer and the institution.^4^^,^^6^–^8^ Three of these four studies occurred in states with multiple tertiary care centers, including Pennsylvania, Massachusetts, Michigan, and Maryland. Compared with these studies, our study found that 23% of the patients had additional cancers diagnosed based on radiologic evaluation by MTB alone.

One possible explanation for the higher percentage of patients with a change in diagnosis is that the MUSC serves as the only NCI-designated cancer center in the state of South Carolina and only one of three academic medical centers in the state. Only 6 of the 70 patients seen had their first opinion at an academic medical center. Additionally, MUSC has the only radiology residency program in the state of South Carolina. As the main tertiary care center in the area, MUSC has a large catchment area from small rural communities lacking in resources and personnel for implementing an MTB.

Using the Health Resources & Services Administration database, we were able to stratify our patient population into lowest and highest health care-funded counties in South Carolina. The state of South Carolina itself is one of the lower-funded states according to this database. Additionally, about 33% of our patient population came from lower-funded counties.^11^ As such, this may mean that implementing MTBs in relatively isolated tertiary care centers that pull from a large population of medically underserved areas has an even more substantial impact on patient care than implementing an MTB in large metropolitan areas with multiple cancer center options.

The most striking finding in this study was that 23% of the patients had an additional cancer found as a result of a second opinion. Of the additional cancers, 14% were in the ipsilateral breast, 1% in the contralateral breast, and 7% in an axillary lymph node. Of the 25 patients who had an additional biopsy, 64% had positive results.

Axillary lymph node status remains an important prognostic factor for patients with breast cancer. Axillary ultrasound is an important tool in the workup for patients with newly diagnosed breast cancer, yet 12 (17%) of the 70 patients presenting for a second opinion had not undergone an axillary ultrasound. By performing axillary ultrasound, we found that 5 (7%) of the 70 patients were node positive. The other 15% of the additional cancers diagnosed were a result of ordering breast ultrasound, MRI, and/or a diagnostic mammogram.

The most important changes in diagnoses found in the pathology review were a change from invasive carcinoma to ductal carcinoma in situ and a change from a breast sarcoma to a malignant phyllodes tumor. Although the pathologists at MUSC are fellowship trained and specialized in breast cancer, one limitation with any change in pathology diagnosis is that tumor behavior clinically is the only way to demonstrate which read is more accurate. Unfortunately, assessment of the clinical course of individual tumors in relation to their pathologic diagnosis fell outside the scope of this review.

Another limitation of this study was that we did not assess whether change in diagnosis resulted in change in management (e.g., breast conservation therapy vs. mastectomy, axillary node dissection vs. sentinel node biopsy, or neoadjuvant vs. adjuvant chemotherapy). We chose not to evaluate change in management because many of the outside records did not clearly state the outside physicians’ plan. In contrast, we could very accurately capture data from the notes of the outside radiologists and pathologists, and could review the record to determine whether genetic counseling had been discussed. Therefore, these were the parameters we chose to compare in our analysis.

## Conclusion

Of the patients presenting for a second opinion reviewed by the MTB in our study, 43% had a change in diagnosis. Most of these changes were a result of additional radiologic recommendations, particularly biopsies. This suggests that review by breast fellowship-trained radiologists on an MTB is one of the areas in which the most benefit can be gained in breast cancer second opinions. The percentage of change in diagnosis resulting from second opinions reviewed at MUSC is higher than in other reported studies. This could be attributed to the high number in our patient population presenting from rural areas, and to the fact that our institution is the only NCI-designated cancer center in the state. Our data provide support for a second opinion by MTBs for patients with breast cancer.
